# Microdialysis of Drug and Drug Metabolite: a Comprehensive *In Vitro* Analysis for Voriconazole and Voriconazole *N*-oxide

**DOI:** 10.1007/s11095-022-03292-0

**Published:** 2022-09-28

**Authors:** Josefine Schulz, Robin Michelet, Markus Zeitlinger, Gerd Mikus, Charlotte Kloft

**Affiliations:** 1 Department of Clinical Pharmacy and Biochemistry, Institute of Pharmacy, Freie Universitaet Berlin, Kelchstraße 31, 12169 Berlin, Germany; 2 Department of Clinical Pharmacology, Medical University of Vienna, Waehringer Guertel 18-20, 1090 Vienna, Austria; 3 Department Clinical Pharmacology and Pharmacoepidemiology, University Hospital Heidelberg, Im Neuenheimer Feld 410, 69120 Heidelberg, Germany

**Keywords:** drug metabolism, exposure, microdialysis, pharmacokinetics, target site, voriconazole

## Abstract

**Purpose:**

Voriconazole is a therapeutically challenging antifungal drug associated with high interindividual pharmacokinetic variability. As a prerequisite to performing clinical trials using the minimally-invasive sampling technique microdialysis, a comprehensive *in vitro* microdialysis characterization of voriconazole (VRC) and its potentially toxic *N*-oxide metabolite (NO) was performed.

**Methods:**

The feasibility of simultaneous microdialysis of VRC and NO was explored *in vitro* by investigating the relative recovery (RR) of both compounds in the absence and presence of the other. The dependency of RR on compound combination, concentration, microdialysis catheter and study day was evaluated and quantified by linear mixed-effects modeling.

**Results:**

Median RR of VRC and NO during individual microdialysis were high (87.6% and 91.1%). During simultaneous microdialysis of VRC and NO, median RR did not change (87.9% and 91.1%). The linear mixed-effects model confirmed the absence of significant differences between RR of VRC and NO during individual and simultaneous microdialysis as well as between the two compounds (p > 0.05). No concentration dependency of RR was found (p = 0.284). The study day was the main source of variability (46.3%) while the microdialysis catheter only had a minor effect (4.33%). VRC retrodialysis proved feasible as catheter calibration for *both* compounds.

**Conclusion:**

These *in vitro* microdialysis results encourage the application of microdialysis in clinical trials to assess target-site concentrations of VRC and NO. This can support the generation of a coherent understanding of VRC pharmacokinetics and its sources of variability. Ultimately, a better understanding of human VRC pharmacokinetics might contribute to the development of personalized dosing strategies.

## INTRODUCTION

Invasive fungal infections are an increasing threat to the global public health causing approximately 1.6 million deaths worldwide every year which is comparable to mortality associated with tuberculosis (1–6). Reasons for this are an expanding susceptible population, e.g. people treated with immunosuppressant drugs, as well as a rise in resistance against antifungal agents (7–10). As the development of new antifungal agents is lagging behind this epidemiological burden (11), one important aspect is the stewardship of existing drugs, such as voriconazole (VRC) (7). VRC is a triazole antifungal agent regularly used in first-line treatment of invasive fungal infections such as aspergillosis and for prophylaxis in immunocompromised patients (12–15). It has been approved in the USA and Europe for almost 20 years (16, 17) and the World Health Organization has classified VRC as an essential medicine (18, 19). Despite the long-term and frequent application in humans, VRC pharmacokinetics (PK) is still not fully understood revealing large intra- and interindividual variability as well as therapy failures and adverse events (20–32). A key source of variability is the extensive and complex metabolism of VRC involving the cytochrome P450 isoenzymes (CYP) 3A4, 2C9 and 2C19 (20). In particular the polymorphic CYP2C19 is catalyzing the formation of voriconazole *N*-oxide (NO), the major circulating metabolite (20, 33–35). NO is considered not to contribute to the antifungal activity of VRC but to induce adverse reactions, in particular photosensitivity and photocarcinogenicity, observed in the context of VRC treatment (36–39). Furthermore, metabolites are also capable of altering the parent’s drug PK, e.g. by inhibition of transporters and enzymes.

PK investigations in clinical trials mainly focus on total drug concentrations determined in plasma. Yet, pathogens usually reside in extravascular spaces, i.e. the interstitial space fluids (ISF), representing the target site for anti-infective drugs (40–43). As plasma and ISF concentrations have been observed to differ extensively (43, 44), regulatory agencies reinforced recommendations to assess target-site concentrations in non-homogenate tissue (45). A powerful tool for this aim is the minimally-invasive sampling technique of microdialysis. In contrast to biopsies (46), it allows continuous sampling over time of the protein-unbound fraction as well as the determination of extracellular concentrations (47–49). For this purpose, the microdialysis catheter is equipped with a selectively-permeable membrane that is inserted into the interstitial space and perfused with a solution (the perfusate) at a flow rate of typically 1–2 µL/min (50, 51). Following the concentration gradient, drug molecules present in the interstitial space diffuse across the membrane into the perfusate which is collected as the so-called microdialysate. As the catheter is continuously perfused, an equilibrium is never reached, resulting in the need to define a relative recovery (RR) value. The RR of a substance describes the fraction of the ISF concentration determined in microdialysate and is required to convert microdialysate concentrations to ISF concentrations. Many calibration methods have been described for the determination of RR, the most frequently used being retrodialysis (47). This approach applies the microdialysis principle in reverse: a minimum 20-fold higher concentration of drug compared to expected ISF concentrations is added to the perfusate (now the so-called retroperfusate) leading to a diffusion of drug molecules into the ISF. Consequently, the decrease in concentration in microdialysate compared to the retroperfusate can be determined and enables the calculation of relative delivery which is assumed to equal RR (47–49).

This microdialysis theory is more complex in a clinical setting (52, 53). During drug therapy not only the drug itself is present in the ISF, but besides endogenous metabolic products, also the drug’s metabolites or concomitantly administered other compounds. Hence, microdialysis investigations might be confounded. Furthermore, often large inter- and intrapatient variabilities are observed in microdialysis trials, potentially deriving from the manually assembled catheters or dissimilar handling procedures during the investigation (44, 52).

Aiming at amalgamating the knowledge on PK of VRC that can be gained by microdialysis investigations and simultaneous drug and metabolite assessment, a comprehensive *in vitro* microdialysis study was performed to address the feasibility of microdialysis sampling of VRC, NO and their combination *in vitro*. Furthermore, potential influential factors of the determination of VRC RR were explored and the performance of VRC retrodialysis for catheter calibration and determination of ISF concentrations of VRC *and* NO evaluated.

## MATERIAL AND METHODS

### Drugs and Materials

*In vitro* investigations were performed with VRC and NO drug substances purchased from Toronto Research Chemicals (Toronto, Canada). For all parts of the study, CMA 60 microdialysis catheters (molar mass cut-off 20 kDa, membrane length 30 mm, M Dialysis AB, Sweden) were used and perfused with Ringer’s solution (B. Braun, Melsungen, Germany). CMA 102 *in vitro* pumps (M Dialysis AB, Sweden) ensured a constant flow of perfusate.

### *In Vitro* Microdialysis Investigations

**Fig. 1:**
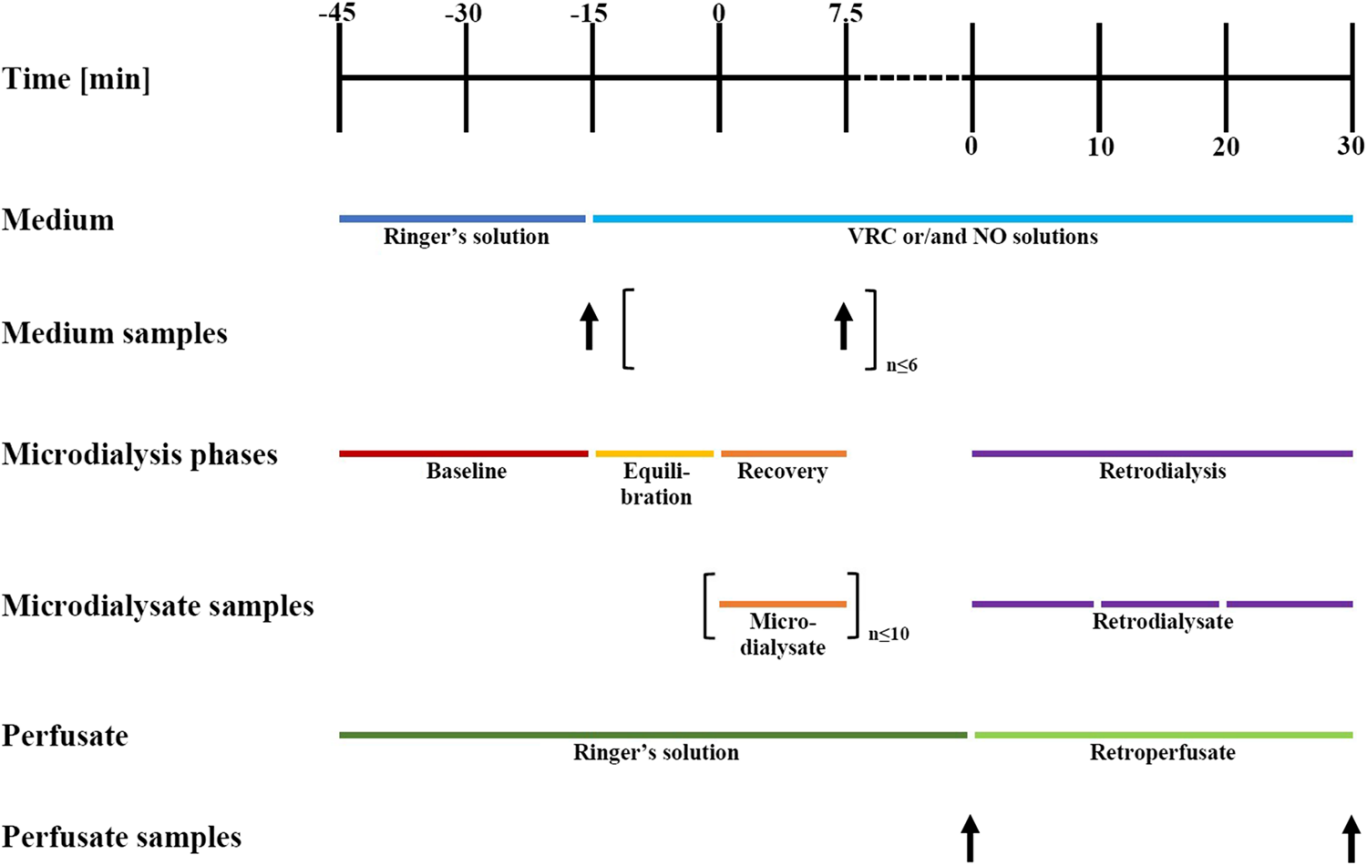
Experimental procedure and sampling schedule for the determination of relative recovery of voriconazole (VRC) and its *N*-oxide metabolite (NO) in a validated *in vitro* microdialysis system including retrodialysis. Times are given relatively to the start of recovery investigations and start of retrodialysis, respectively.

A previously developed and validated *in vitro* microdialysis system (IVMS), consisting of a pump, catheter, thermos and stirring module, was used to perform all experiments in a standardized way (54, 55). The IVMS enabled the parallel use of up to four microdialysis catheters and ensured consistent conditions of 37 °C in the catheter-surrounding medium. The medium was constantly stirred to ensure unhindered diffusion across the microdialysis membrane. Analyte-free Ringer’s solution was used as perfusate with a flow rate of 2 µL/min. Hence, *in vitro* applied conditions and parameters mimicked closely the *in vivo* situation during clinical microdialysis applications (51, 56). The catheter-surrounding medium, mimicking the ISF, consisted of Ringer’s solution spiked with VRC, NO or a mixture of both. All *in vitro* experiments were performed according to a fixed schedule, starting with catheters being flushed and equilibrated before investigations of RR began (Fig. 1). All microdialysis catheters were reused in several experiments, hence, after each experiment, the catheters were flushed with MilliQ^®^ water. Afterwards, the inlet and outlet tubings were sealed, the membrane covered and the catheter stored in a light-excluding space at room temperature. Overall, investigations were performed in a consecutive way, starting with microdialysis of VRC at low concentrations only, before proceeding with NO, the combination and a larger concentration range. In the following presented analysis, results from all performed investigations were pooled to obtain the largest possible data base. However, as a consequence of the stepwise approach, some fluctuations between the total number of samples, the study days and applied microdialysis catheters exist between VRC and NO. In total, on seven days investigations were performed, assessing individual microdialysis of VRC and its *N*-oxide metabolite as well as simultaneous sampling. Therefore, data from four different scenarios was available: VRC, NO, VRC + NO and NO + VRC.

#### Relative Recovery of Voriconazole and its *N*-oxide Metabolite During Individual Microdialysis

The RR of VRC and NO in the absence of the respective other was investigated to assess the initial microdialysis behavior as a prerequisite for a comparison to the more realistic situation of simultaneous microdialysis of VRC and NO. Therefore, five concentrations (0.01, 0.1, 0.2, 0.5 and 3 µg/mL) for VRC and four (0.01, 0.1, 0.5 and 3 µg/mL) for NO in the catheter-surrounding medium were used. For VRC, individual microdialysis investigations were performed on five study days using five different microdialysis catheters and for NO on three days using four catheters. Microdialysate samples were collected over a period of 7.5 min with up to ten intervals consecutively on one study day with the same catheter. Additionally, the catheter-surrounding medium was sampled up to six times throughout the experiment (Fig. 1). RR was assessed as ratio of the individual microdialysate concentration (C_µD_) and mean medium concentration (C_Medium_) (Eq. 1).

1 $$RR,\%=\frac{C_{\mu D}}{C_{Medium}}\cdot100\%$$

RR values were graphically explored to evaluate their dependency on concentration, the individual microdialysis catheter and the study day to assess the impact of experimental factors on *in vitro* recovery investigations. Thereby, the respective other factors were pooled.

#### Relative Recovery of Voriconazole and its *N*-oxide Metabolite During Simultaneous Microdialysis

Simultaneous microdialysis of VRC and NO was investigated using four catheters on three study days. In total, five different solutions combining VRC + NO were examined: 0.01 + 0.01, 0.2 + 0.5, 0.5 + 0.5, 3 + 0.01 and 3 + 3 µg/mL, respectively. Combinations of high NO and low VRC concentrations were not feasible due to the impurity of the NO reference standard containing approximately 2% of VRC. Medium samples were taken throughout each experiment and used to calculate RR according to Eq. 1. The obtained RR values were evaluated graphically to assess changes in RR compared to individual microdialysis. Additionally, the influence of the concomitant concentration of the second compound was explored.

#### Linear Mixed-Effects Modelling

For a comprehensive evaluation, a linear mixed-effects (LME) model was built to *simultaneously* assess the impact of experimental factors on RR as well as to explore the difference between RR of VRC, NO and their combination. Investigated experimental factors comprised the VRC and NO concentration, the used individual microdialysis catheter and the respective study day. The RR values of VRC and NO were set as dependent variable and the nominal concentration (C_Nominal_) as well as the scenario were defined as fixed terms. In total, RR corresponded to four different scenarios, which were integrated in the model as categorical variables.

RR of the individual microdialysis of VRC was thereby the reference scenario and estimated as the coefficient a_0_ corresponding to the y-intercept of the linear model. The RR of the other three scenarios was estimated as absolute deviation from RR of VRC as coefficient a_1_ for each of the remaining three scenarios. The slope of the linear regression for each scenario was described by the impact of C_Nominal_ and its coefficient a_2_. Furthermore, the individual microdialysis catheter (*n* = 5) and the study day (*n* = 7) were defined in the model as crossed random effects (η) as they were assumed to contribute to the observed variability, whereas ɛ depicted the remaining unexplained variability (Eq. 2).

2 $$\text{RR}=a_0\text{+}a_1\cdot\text{Scenario}+a_2\cdot \mathrm C_{Nominal}\text{+}\eta(C\text{atheter)+}\eta\text{(Study day}{)+{\epsilon}}$$

The estimates were obtained by the restricted maximum likelihood criterion applying the “lmer” function of the lme4 package (57) in the software R (version 3.6.0) (58). All fixed effects were tested for statistical significance by applying an F-statistic using Satterthwaite's approximation for the degrees of freedom, using the lmerTest package (59) in R (58). The result was considered statistically significant with 95% confidence intervals (CI) excluding zero and p-values ≤ 0.05.

#### *In Vitro* Retrodialysis

VRC retrodialysis was performed by exchanging the perfusate to retroperfusate that consisted of Ringer’s solution spiked with VRC at a concentration of 60 µg/mL. Three retrodialysate samples were collected per microdialysis catheter for 10 min each while retroperfusate samples (*n* = 3) were taken at the beginning and the end of retrodialysis (Fig. 1). Relative delivery (RD) was calculated as 100% minus the ratio of the VRC concentration in retrodialysate (C_Retrodialysate_) and the concentration in retroperfusate (C_Retroperfusate_, Eq. 3) and used to calculate the concentration of VRC and NO in the catheter-surrounding medium (Eq. 4).

3 $$RD,\%=100-\left(\frac{C_{Retrodialysate}}{C_{Retroperfusate}}\right)\cdot100\%$$

4 $$C_{Medium}=\frac{C_{\mu D}}{RD,\%}\cdot100\%$$

The performance of VRC retrodialysis was assessed by comparing these calculated VRC and NO concentrations to the direct measurements in the catheter-surrounding medium (*n* = 6).

### Bioanalysis

Quantification of VRC and NO in microdialysate was performed using an LC–MS/MS assay validated according to the EMA guideline on bioanalytical method validation (60, 61). Briefly, an Agilent 1290 Infinity II LC system combined with an InfinityLab Poroshell 120 Phenyl Hexyl column (RP, 2.1 × 100 mm, 2.7 µm, Agilent Technologies, Waldbronn, Germany) were applied for chromatography. A gradient method of methanol and ultrapure water (both with 0.1% [V/V] formic acid) at a flow rate of 0.350 mL/min ensured chromatographic separation. The Agilent triple quadrupole MS/MS system (G6495A) used an electrospray ionization source operated in positive ion mode and monitored for quantification the transition of *m/z 350 → 281* for VRC, *m/z 366 → 224* for NO and *m/z 285 → 193* for the internal standard, diazepam. The calibration range for VRC and NO was 0.004–4 µg/mL for microdialysate, using only 5 µL of sample volume. Performance of the analysis was controlled in all analytical runs by evaluation of separately prepared quality control samples and met the acceptance criteria set by the EMA guideline with accuracies within 100% ± 15% (± 20% at the lower limit of quantification) and precision ≤ 15% coefficient of variation (≤ 20% at the lower limit of quantification) (60).

## Results

### Relative Recovery of Voriconazole and its *N*-oxide Metabolite During Individual Microdialysis

First, RR of VRC and its *N*-oxide metabolite was explored in the absence of the respective other. Overall, both analytes revealed high and consistent RR values. Pooling all RR values of VRC across all concentrations, study days and used microdialysis catheters, the median RR was 87.6% (95% CI: 86.5% – 88.8%, *n* = 114). The observed minimum and maximum RR values were 77.4% and 101%, respectively. The median RR of NO across all concentrations, study days and microdialysis catheters was high and exceeded with 91.1% (95% CI: 88.4% – 94.5%, *n* = 85) the median RR of VRC by an absolute difference of 3.5%. Minimum and maximum observed RR values for NO were 79.0% and 105%, respectively.

#### Relative Recovery of Voriconazole and its *N*-oxide Metabolite in Dependence of Concentration 

**Fig. 2:**
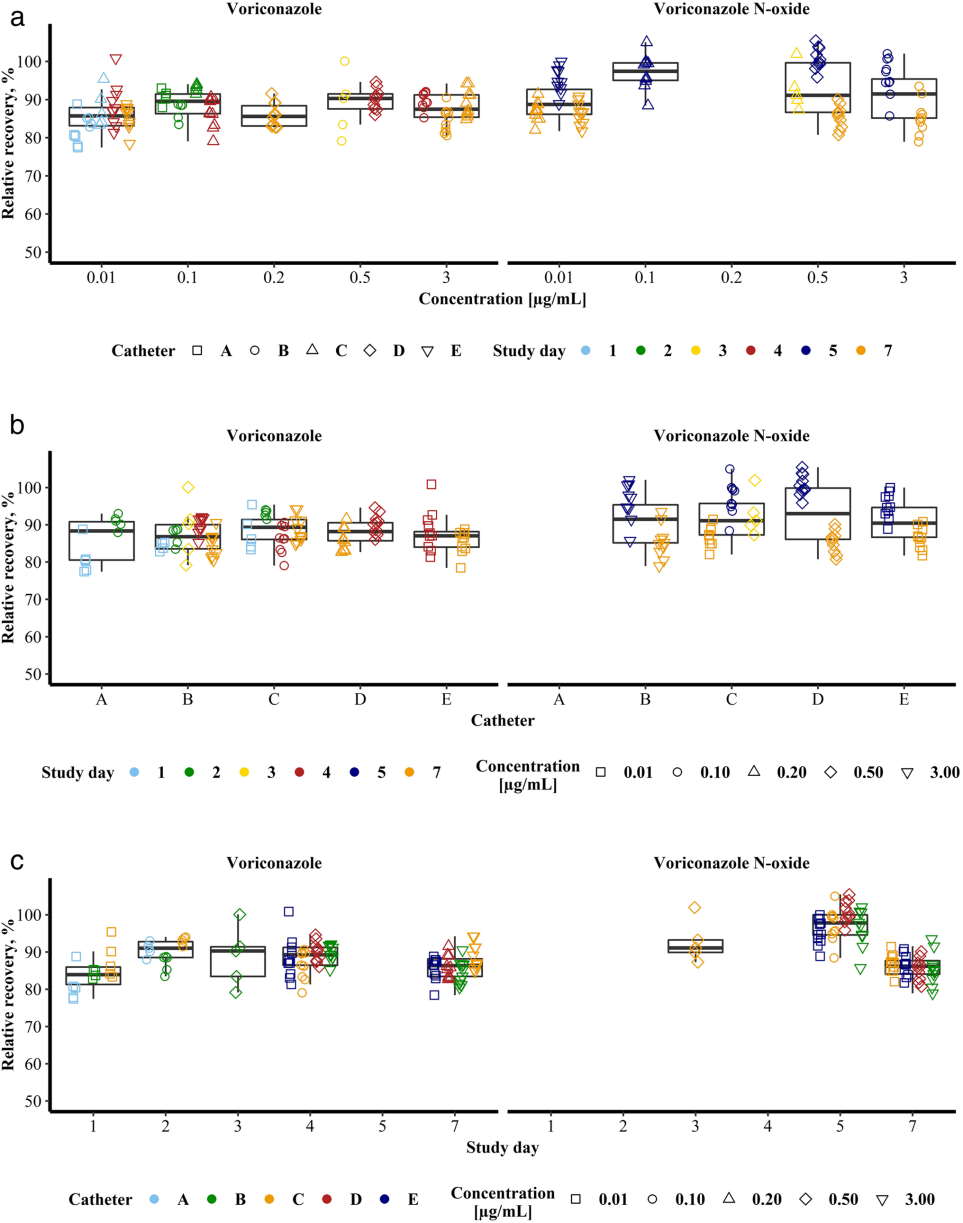
* In vitro* relative recoveries in percent of voriconazole (left panels) and its *N*-oxide metabolite (right panels) in dependency of the nominal concentration (**a**), the microdialysis catheter used (**b**) and the study day (**c**). The boxes represent the interquartile ranges (IQR) including the respective median of all data points as a bold line. The upper whiskers extend to the largest value but no further than 1.5 * IQR, the lower whiskers extend to the smallest value at most 1.5 * IQR. Individual data points of relative recovery are overlaid (*n* = 114 for voriconazole and *n* = 85 for voriconazole *N*-oxide) as determined from individual microdialysis concentrations and mean concentrations determined in catheter-surrounding medium.

The median RR of VRC in function of its concentration from 0.01 to 3 µg/mL ranged from 85.6% (95% CI: 82.8% – 89.2%) at 0.20 µg/mL (*n* = 10) to 90.3% (95% CI: 87.4% – 91.5%) at 0.50 µg/mL (*n* = 15). Thus, no concentration dependence was observable as CI were overlapping. Moreover, no tendencies, i.e. de- or increasing RR with increasing VRC concentrations were observable (Fig. 2a, left). The variability of RR of VRC at the individual concentrations was comparably small indicated by interquartile ranges (IQR) of a minimum of 3.93% points at a concentration of 0.50 µg/mL (*n* = 15) and a maximum of 5.86% points at a concentration of 3 µg/mL (*n* = 30).

The lowest median RR for NO was observed at the lowest concentration of 0.01 µg/mL with 88.8% (95% CI: 86.7% – 91.5%, *n* = 30) and the highest with 97.4% (95% CI: 93.7% – 100%) at the second lowest concentration of 0.10 µg/mL (*n* = 10). Although CI were not overlapping, further evaluations were needed to assess the significance of this observation (see 3.3 Linear mixed-effects modelling), as all RR values at the concentration of 0.10 µg/mL derived from the same study day and the same microdialysis catheter. Moreover, there was no continuing trend for in- or decreasing RR with increasing NO concentrations observable (Fig. 2a, right). The observed variability in RR was larger for NO than for VRC as indicated by an IQR of a minimum of 4.57% points at a concentration of 0.10 µg/mL (*n* = 10) and a maximum of 13.0% points at a concentration of 0.50 µg/mL (*n* = 25).

#### Relative Recovery of Voriconazole and its *N*-oxide Metabolite in Dependence of the Microdialysis Catheter

For VRC, a minor influence of the used microdialysis catheter on RR was observable. Median RR were determined for every catheter, pooling data from different study days and concentrations. The catheter number was thereby allocated randomly not allowing to draw conclusions about tendencies. Median RR of VRC were 88.4% (95% CI: 77.9% – 91.6%, *n* = 10), 86.8% (95% CI: 85.1% – 89.2%, *n* = 34), 89.3% (95% CI: 86.2% – 91.2%, *n* = 30), 88.2% (95% CI: 85.9% – 90.4%, *n* = 20) and 87.1% (95% CI: 84.1% – 87.9%, *n* = 20) for the catheters A to E, respectively (Fig. 2b, left). Thus, the maximum absolute detected difference in median RR between catheters was 2.5% points indicating a minor influence of the individual microdialysis catheter. Also the intracatheter variability of RR was mostly comparable with absolute IQR ranging from 4.15% (catheter E, *n* = 20) to 6.50% (catheter B, *n* = 34), with the exception of catheter A with an IQR of 10.3% (*n* = 10).

For NO the observations were comparable. Here, median RR ranged from 90.5% in catheter E (n = 20) to 93.0% in catheter D (*n* = 20) (Fig. 2b, right), resulting in a maximum absolute difference in RR of 2.5%. Although the same microdialysis catheters were used as for VRC, intercatheter variability of RR was increased for NO with IQR of 10.2%, 8.45%, 13.7% and 7.99% points for the catheters B to E, respectively.

#### Relative Recovery of Voriconazole and its *N*-oxide Metabolite in Dependence of the Study Day

Overall, the study day had the largest influence on VRC and NO RR. VRC RR was investigated on study days 1, 2, 3, 4 and 7 and resulted in median RR values of 83.9% (95% CI: 80.5% – 88.8%, *n* = 14), 91.1% (95% CI: 88.5% – 93.0%, *n* = 15), 90.3% (95% CI: not applicable as *n* = 5), 89.3% (95% CI: 87.4% – 90.4%, *n* = 40) and 86.4% (95% CI: 84.8% – 87.1%, *n* = 40) on the respective day (Fig. 2c, left). Consequently, the maximum observed absolute difference in RR between study days was 7.2%. However, within one study day, RR of VRC was relatively constant and independent of catheter and VRC concentration.

RR of NO was investigated on study days 3, 5 and 7 and fluctuated with median RR of NO of 91.1% (95% CI: not applicable as *n* = 5), 97.8% (95% CI: 94.9% – 99.5%, *n* = 40) and 86.2% (95% CI: 85.0% – 87.1%, *n* = 40), respectively, more than RR of VRC (Fig. 2c, right). In particular the absolute difference of 11.6% in RR of NO between study day 5 and 7 was prominent. In particular as the variability was comparable with IQR of 3.36%, 5.39% and 3.61% points on study day 3, 5 and 7, respectively.

### Relative Recovery of Voriconazole and its *N*-oxide Metabolite During Simultaneous Microdialysis

**Fig. 3:**
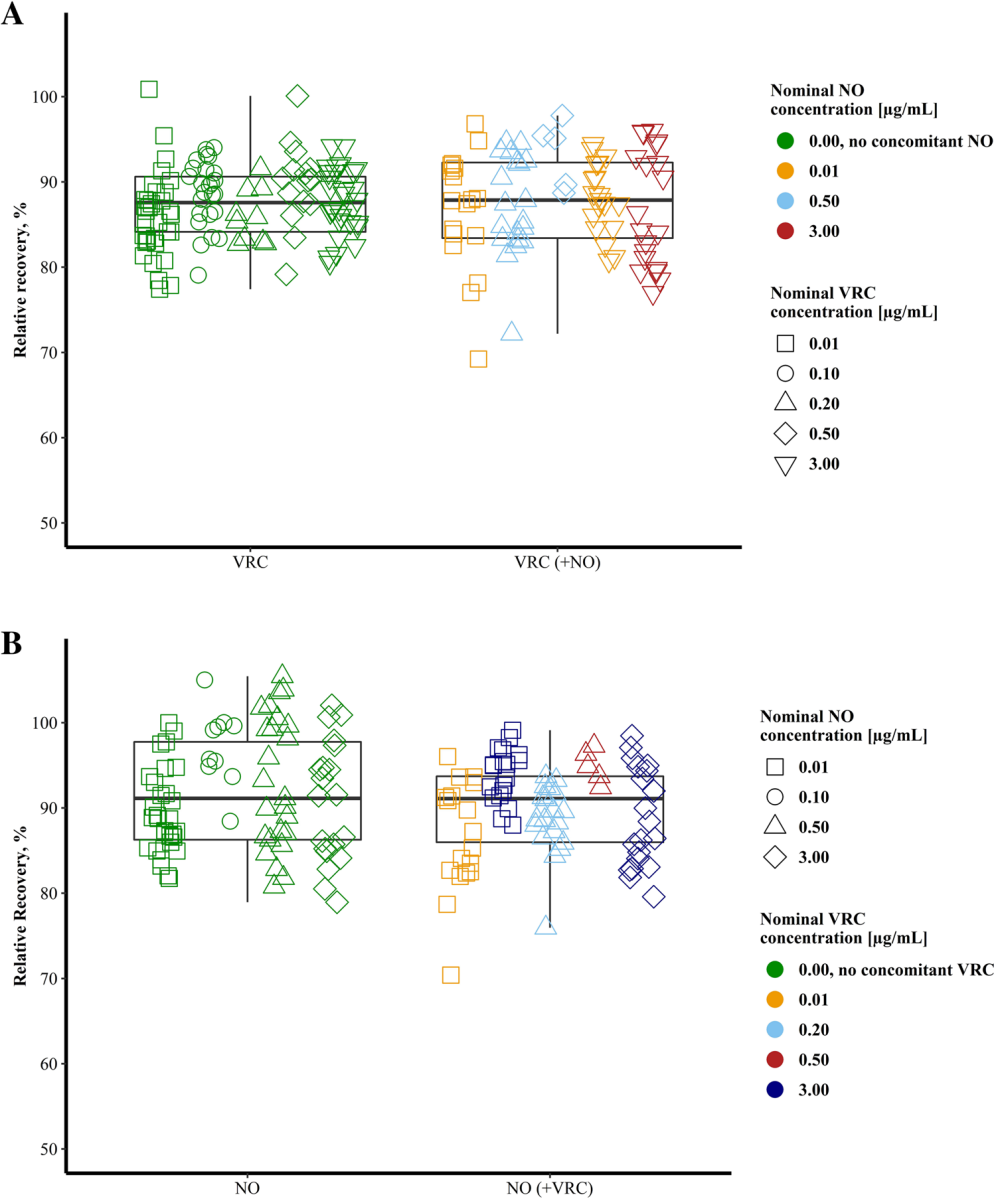
*In vitro* relative recovery of voriconazole (VRC, A) and voriconazole *N*-oxide (NO, B) in percent in the absence (left panel) and presence (right panel) of the respective other.

In a second step, RR of VRC and its *N*-oxide metabolite were investigated in the presence of varying concentrations of the respective other. Pooling all data from different concentrations, catheters and study days, median RR of VRC in the presence of NO was 87.9% (95% CI: 85.3% – 90.5%, *n* = 82). This was comparable to an absolute change of RR of 0.3% compared to RR determined in the absence of NO (Fig. 3A). Furthermore, not only the mere presence of NO was taken into consideration but also the respective concomitant NO concentration. An absolute decrease in VRC RR of 2.5% was observed when the combination of 0.01 µg/mL VRC plus 0.01 µg/mL NO was compared to the combination of 3 µg/mL VRC plus 3 µg/mL NO. This difference was classified as minor, as also CI were widely overlapping (Table I).

**Table I:** Median relative recovery of Voriconazole and Voriconazole *N*-Oxide during simultaneous microdialysis of five different concentration combinations

Concentration [µg/mL]		Median relative recovery voriconazole, % (95% confidence interval)	Median relative recovery voriconazole *N*-oxide, % (95% confidence interval)	n
Voriconazole	Voriconazole *N*-oxide			
0.01	0.01	87.9 (83.7 – 91.6)	86.3 (82.5 – 91.4)	18
0.20	0.50	86.4 (83.3 – 92.4)	89.1 (87.3 – 91.3)	20
0.50	0.50	95.1 (n.a.)	94.9 (n.a.)	5
3.0	0.01	87.9 (84.7 – 92.2)	95.0 (91.4 – 96.3)	19
3.0	3.0	85.4 (81.1 – 92.9)	87.4 (84.1 – 94.4)	20

n.a., not applicable due to low number of replicates

For NO, pooled data across all concentrations, microdialysis catheters and study days, median RR in the presence of VRC resulted in a RR of 91.1% (95% CI: 88.6% – 92.4%, *n* = 82) and hence no shift was observed compared to individual determinations in individual microdialysis investigations (Fig. 3B). The concomitant VRC concentration did not have a large impact on RR of NO. A difference in absolute RR of NO of + 1.1% was observed when comparing the combination of 0.01 and 0.01 µg/mL to the combination of 3.0 and 3.0 µg/mL VRC and NO (Table I). The combination of a high VRC concentration (3.0 µg/mL) with a low NO concentration (0.01 µg/mL) led to a slightly increased RR of NO of 95.0%. However, CI were still overlapping indicating a negligible effect (Table I).

### Linear Mixed-Effects Modelling

All exploratory and graphical assessments were confirmed and furthermore quantified by an LME model enabling the *simultaneous* consideration of all potentially influencing factors. In this model, RR of VRC in the absence of NO was set as the reference scenario which resulted in an estimated RR of 89.7% (a_0_, Table II). No significant influence of concentration was revealed within the investigated concentration range with a small a_2_ value and its 95% confidence interval including zero (p ≥ 0.05, Table II). Moreover, the absolute deviations in RR of the other three scenarios in comparison to RR of VRC individual microdialysis were not significant with 95% CI including zero and p-values > 0.05 (Table II). The random effects could explain in total 50.6% of the observed variability. Here, the study day was the most important factor, which explained a standard deviation of 4.10% (95% CI: 2.31% – 7.30%) points on RR and had a share of 46.3% in the total variability. In contrast, the individual microdialysis catheter only entailed an absolute variability of 1.25% (standard deviation, 95% CI: 0.481% – 3.19%) and caused thus 4.33% of the total variability. A further standard deviation of 4.23% (95% CI: 3.92% – 4.54%) points remained unexplained which equaled 49.4% of the total variability in the data (Table II).

**Table II:** Parameter estimates for the fixed and random effects of the linear mixed-effects model evaluating the dependence of the *in vitro* relative recovery (RR) on the nominal concentration and the investigated scenario, i.e. Voriconazole (VRC) and its *N*-Oxide Metabolite (NO) in the absence of the respective other and the combination

Parameter	Estimate	95% confidence interval of the estimate	P-value
$${a}_{0}$$			
RR of VRC	89.7%	86.2% – 93.3%	1.8∙10^–11^
$${a}_{1}$$			
ΔRR of VRC + NO	-1.56%	-3.18% – + 0.113%	0.0636
ΔRR of NO	0.473%	-1.24% – + 2.28%	0.5936
ΔRR of NO + VRC	0.868%	-0.757% – + 2.54%	0.3014
${a}_{2}$	0.309 mL/µg	-0.254 – + 0.900 mL/µg	0.2840
η (Study day)*	4.10%	2.31% – 7.30%	n.a
η (Catheter)*	1.25%	0.481% – 3.19%	n.a
ε (residual)*	4.23%	3.92% – 4.54%	n.a

a_0_: intercept of the linear regression corresponding to the estimated RR of voriconazole

a_1_: absolute deviation in RR for the three scenarios

a_2_: slope of the linear regression, i.e. change of RR in dependence on concentration

η, ε: random effect variables

^*^: reported as standard deviation

n.a.: not applicable

### *In Vitro* Retrodialysis

Concentrations of VRC and NO in catheter-surrounding medium were determined by direct measurement as well as by VRC retrodialysis. At a nominal concentration of 0.01 µg/mL VRC, median concentrations in catheter-surrounding medium determined by retrodialysis (*n* = 8–10 each) were at the two days of investigation -12.8% and -9.2% lower than the median of directly measured concentrations (*n* = 6 each). At a concentration of 0.02 µg/mL (*n* = 10 each retrodialysis, *n* = 6 each direct measurement) the deviation was -13.8% and -4.2%. For higher concentrations of VRC (3 µg/mL) four investigations were performed and resulted in deviations of -1.6%, + 1.6%, -7.1% and -12.9% (*n* = 9–10 each retrodialysis, *n* = 6 each direct measurement) respectively.

Also for NO, retrodialysis of the parent compound VRC was applied. At a nominal concentration of 0.01 µg/mL NO, median concentrations in catheter-surrounding medium determined by retrodialysis (*n* = 8–10 each) were at the four investigation -14.9%, -3.0%, -5.0% and -1.5% lower than the median of directly measured concentrations (*n* = 6 each). The deviation at a concentration of 0.50 µg/mL NO was -5.9% and -4.6% at the two study days (*n* = 9–10 each retrodialysis, *n* = 6 each direct measurement), respectively. Lastly, at a nominal concentration of 3.0 µg/mL NO retrodialysis resulted in median concentrations of -9.7% and + 0.4% compared to direct measurements (*n* = 10 each retrodialysis, *n* = 6 each direct measurement).

## DISCUSSION

Our comprehensive *in vitro* analysis demonstrated the feasibility of the simultaneous microdialysis of VRC and its major circulating metabolite NO as would be the case in a patient during VRC treatment. Secondly, we identified potential sources for the variability regularly observed in microdialysis data. Thirdly, we present a convenient approach for the simultaneous catheter calibration for VRC and NO. Microdialysis is a valuable tool for the assessment of unbound drug concentrations continuously over time in ISF, i.e. the target site of the infection. However, for the design and execution of high quality clinical microdialysis trials knowledge of the behavior of the drug in microdialysis sampling is essential. Consequently, reliable results and derivation of correct conclusions in clinical trials rely on a thorough *in vitro* characterization (49). The presented *in vitro* investigations revealed high RR of 87.6% of VRC and confirmed the previously reported VRC RR of 91.5% as well as its independence of concentration (54). Additionally, these observations could be extended to 100-fold lower VRC concentrations, covering a more clinically relevant range (51). This consistency of RR demonstrated an unhindered diffusion of drug molecules across the catheter membrane and thus was the foundation of reliable results. However, in *in vivo* applications by nature various substances are simultaneously present in the ISF, potentially interacting, e.g. by competition for the diffusion across the microdialysis membrane which might have an impact on RR. In the context of drug therapy this might be the case for other concomitantly administered drugs (62) but also the drug’s metabolites. Therefore, feasibility of simultaneous microdialysis sampling of VRC and its major metabolite, NO, was to be assessed. The slightly more hydrophilic metabolite does not differ largely from VRC in terms of molecular mass (349 *versus* 365 g/mol), providing good reason for the very similar behavior in microdialysis (33). RR of NO was also high with 91.1% and independent of concentration when sampled individually. The combination of VRC and NO in *in vitro* microdialysis did not lead to changes in their RR, again demonstrating their unsaturated and unhindered diffusion. However, graphical evaluations suggested an impact of experimental factors, i.e. the microdialysis catheter and the study day, as RR values derived consecutively on the same study day with the same catheter were clustered around one central value. Thus, by mere statistical testing between data subsets, using e.g. t-tests, results could be confounded by experimental factors, potentially resulting in false-positive significant differences. Therefore, to allow for an unbiased analysis of the obtained data, a joint statistical evaluation of potentially impacting experimental factors, compound concentration and scenarios was performed applying an LME model approach. By implementing the study day and the catheter as random factors in the model, their variability was taken into account when evaluating the significance of the fixed effects (concentration and scenario). Our analyses demonstrated that RR of VRC and its *N*-oxide metabolite in the absence of the respective other was not significantly different to RR determined in the presence of the respective other. Furthermore, no significant differences between VRC and NO RR were detected. Instead, experimental factors were revealed as sources of variability. On the one hand, although microdialysis catheters are assembled by hand, no major variability originated from there, which was in agreement with a clinical trial investigating RR of several antiinfectives (52). On the other hand, the study day played a large role. As a strict protocol was followed, this was unexpected and might originate from further unidentified factors, e.g. instruments such as the microdialysis pumps causing fluctuations in the flow rate. Potentially, also the storage of the microdialysis catheters could have played a role. A damage of the catheter membrane was unlikely as it results in most cases in a considerably reduced volume of microdialysate, which was not observed. Furthermore, if membrane characteristics were altered by continued storage, a trend of de- or increasing RR values would have been expected, which was also not observed in the present study. Lastly, air pockets might have formed during storage depicting a potential diffusion barrier (62) although the equilibration procedure aimed at their removal. The analysis of clinical microdialysis data revealed interindividual variability as an important source of variability (52), potentially due to the specific catheter location in the tissue between individuals. Hence, *in vitro* as well as *in vivo* investigations might benefit from a standardized, reproducible handling of catheter administration. In our study, almost 50% of variability in the data remained unexplained (being equal to a standard deviation of 4.23% points) but are likely to include intracatheter variability as well as bioanalytical imprecision which is allowed to amount to 15% coefficient of variation (60, 63). A further extension or adaptation of the LME model by inclusion of the concomitant concentration of the combination partner was not possible due to overparameterization. Consequently, more experiments would be needed. However, based on the exploratory analyses an influence is unlikely.

Retrodialysis is commonly performed substance-specific for individual catheter calibration *in vivo* (64). Yet, metabolites are to be considered as chemical substances that are not licensed for the use in humans and hence are not allowed as supplement in retroperfusate solutions. Consequently, a different approach for the determination of RR was required. The result, that the RR values of VRC and NO were not significantly different *in vitro* allowed for the hypothesis of using VRC retrodialysis for both the parent compound and its metabolite. The feasibility of this hypothesis was confirmed *in vitro* resulting in marginal deviations between VRC and NO. However, overall retrodialysis rather overestimated RR for both substances, resulting in an underestimation of concentrations in the catheter-surrounding medium with a deviation of + 1.6% to -14.9% compared to directly measured concentrations. As previous investigations demonstrated the conformity of VRC RR and rD in CMA 60 catheters (54), relevant unspecific binding of VRC or NO to components of the microdialysis catheter was excluded as a cause for the observed deviation. Thus, overall a slight imprecision in the method itself was assumed. In context of the large total variability often observed in clinical microdialysis trials this might be negligible and the use of retrodialysis still more convenient and the obtained results more accurate and precise compared to other catheter calibration procedures (47). Nevertheless, further refinement of existing catheter calibration methods to derive tissue fluid concentrations is desirable. An interesting new approach applied a calibrator in perfusate observing simultaneously the loss of the calibrator and recovery of the drug of interest saving the time and effort of retrodialysis altogether (65, 66). Either way, individual *in vivo* relative recovery determinations are essential and a transfer of *in vitro* RR data insufficient as the presence of cells and extracellular matrix leads to a different movement of molecules, i.e. tortuosity (47, 49). This results in lower total RR *in vivo* than *in vitro*. For VRC this observation was confirmed, although not very distinct, with *in vivo* RR of 84.9% and 85.2% (51, 56) compared to 87.6% *in vitro*. Nevertheless, our findings of identical behavior and comparable RR of VRC and NO *in vitro* are assumed to be applicable also to the *in vivo* situation, justifying the utilization of VRC retrodialysis for determination of tissue fluid concentrations of NO.

Overall, we laid the foundation for the clinical application of the simultaneous microdialysis of VRC and its *N*-oxide metabolite. The presented comprehensive *in vitro* investigation demonstrated that VRC and its *N*-oxide metabolite can be sampled by microdialysis and that RR is not impacted by the concentration of the two compounds. This is an essential finding as VRC is metabolized mainly by the polymorphic CYP2C19 (67), hence it can be assumed that *in vivo* variability of VRC to NO concentrations between different CYP2C19 genotypes or even in the same individual will not influence the respective RR. Therefore, also the application of retrodialysis as a catheter calibration method is applicable at all. Moreover, using only the parent compound, i.e. VRC, to derive ISF concentrations of the parent and the metabolite, represents a distinct advantage for the clinical application and further research.

Although NO is not contributing to the antifungal activity of VRC, it has been suspected to contribute to adverse events, in particular to the emergence of phototoxicity and photocarcinogenicity occurring in patients with long-term VRC treatment (36–39). A plausible mechanism has been presented based on *in vitro* investigations (39). Thus, Therapeutic Drug Monitoring and individualized dosing strategies might not only need to target certain VRC concentrations, but also limit NO concentrations (68). Here, target-site exposures of VRC and NO, instead of total plasma concentrations, might yield more informative relationships between VRC PK and pharmacodynamics to guide the optimization of VRC dosing regimen (69).

## CONCLUSION

Microdialysis is a powerful method for the determination of unbound concentrations in ISF, i.e. the target site of antifungals such as VRC. Here, we demonstrated that comprehensive *in vitro* investigations are not only important components but necessary prerequisites on the path to meaningful clinical microdialysis trials. We demonstrated the feasibility of simultaneous microdialysis of VRC and its major metabolite in clinically relevant concentrations as well as the applicability of VRC retrodialysis for both, which is the essential framework for reliable *in vivo* investigations. The assessment of ISF as well as metabolite concentrations can particularly increase the knowledge of the PK of a drug, demonstrate the full extent of interindividual variability as well as its sources. Further research is required to investigate VRC and NO ISF concentrations *in vivo*. In particular, PK in dependence of the CYP2C19 metabolizer status should be assessed. In perspective, the elucidation of human VRC PK will guide the development of personalized dosing regimen for VRC.

## Data Availability

The datasets generated during and/or analyzed during the current study are available from the corresponding author on reasonable request.
